# The Earth Microbiome Project: Meeting report of the “1^st^ EMP meeting on sample selection and acquisition” at Argonne National Laboratory October 6^th^ 2010.

**DOI:** 10.4056/aigs.1443528

**Published:** 2010-12-25

**Authors:** Jack A. Gilbert, Folker Meyer, Janet Jansson, Jeff Gordon, Norman Pace, James Tiedje, Ruth Ley, Noah Fierer, Dawn Field, Nikos Kyrpides, Frank-Oliver Glöckner, Hans-Peter Klenk, K. Eric Wommack, Elizabeth Glass, Kathryn Docherty, Rachel Gallery, Rick Stevens, Rob Knight

**Affiliations:** 1 Argonne National Laboratory, Argonne, IL USA; 2 Department of Ecology and Evolution, University of Chicago, Chicago, IL USA; 3 Computation Institute, University of Chicago, Chicago, IL USA; 4 Lawrence Berkeley National Laboratory, Earth Sciences Division Berkeley, CA USA; 5 Center for Genome Sciences & Systems Biology, St. Louis, MO USA; 6 Department of Chemistry and Biochemistry, UCB Boulder, CO USA; 7 Center for Microbial Ecology, Michigan State University, East Lansing, MI USA; 8 Department of Microbiology, Cornell University, Ithaca, NY USA; 9 NERC Centre for Ecology & Hydrology, Oxford, U.K.; 10 DOE Joint Genome Institute, Walnut Creek, CA USA; 11 MPI for Marine Microbiology, Bremen, Germany; 12 DSMZ - Deutsche Sammlung von Mikroorganismen und Zellkulturen GmbH, Braunschweig, Germany.; 13 University of Delaware, Delaware Biotechnology Institute, Newark, DE USA; 14 NEON, Boulder, CO USA

## Abstract

This report details the outcome the first meeting of the Earth Microbiome Project to discuss sample selection and acquisition. The meeting, held at the Argonne National Laboratory on Wednesday October 6^th^ 2010, focused on discussion of how to prioritize environmental samples for sequencing and metagenomic analysis as part of the global effort of the EMP to systematically determine the functional and phylogenetic diversity of microbial communities across the world.

## Introduction

Understanding microbes (bacterial, archaeal, eukaryal and viral) in terms of who they are and what they do is the challenge of microbial ecology. This concept was explored and a conceptual framework and action plan sketched out at the Terabase Metagenomics Workshop held in Snowbird, Utah between 18th and 24th July 2010; it was at this meeting that concept of the Earth Microbiome Project was initiated [1]. The Earth Microbiome Project (EMP) presents a revolution in how we tackle this problem and defines both questions and a potential suite of tools to provide answers. The EMP will provide a quantum leap in our ability to interrogate ecosystem-scale microbial ecology through a truly global collaborative project. Earth sustains a standing population of approximately 1 x 10^30^ microbial cells. To date, total available environmental DNA sequence data (from metagenomic studies) constitutes significantly less than 1% of the total DNA found in a liter of seawater or a gram of soil. Hence, we have vastly under sampled the complexity and diversity microbes on this planet. However, recent advances in high-throughput sequencing technologies have provided an unprecedented opportunity to explore the microbial universe, and we propose to leverage this capability at a scale many orders of magnitude greater than any previously conceived study. We wish to sequence microbes and microbial communities from every conceivable biome.

**By exploring genetic information from every ecosystem we hope to achieve three main goals:**
1. Attempt to produce a complete inventory of protein diversity.
2. Define microbial community structure across microbial ecosystems and explore at different scales what structures it, i.e. defining microbes in environmental parameter space – a microbe centric view.
3. Creation of a global database of samples, genes and proteins the can be used to answer fundamental questions about the ecology of life on and off the earth.

Microbes are the life-support system for our planet. Without them there could be no other life—and yet we know little of the mechanistic details by which microbial communities provide this support. We as a species now have a significant impact on this planet, for example, we are changing weather systems, altering the chemistry of the atmosphere and acidifying the oceans. These large-scale changes will affect microbial life and, with it, all life on Earth. It is therefore essential that we, the scientific community, develop a strategy to improve our understanding of the role and importance of microbes and in turn how microbes will respond to anthropogenic forces within ecosystems.

The EMP aims to select and acquire 100,000- 200,000 samples from numerous and diverse environments across the world that will support large-scale modeling efforts aimed at understanding how changes in microbial communities relate across different spatial and temporal scales. The bottlenecks for this project will likely not be sequencing, but rather identifying projects that can provide samples, determining whether the samples adhere to strict requirements for associated metadata that support integration efforts, and the infrastructure, protocol and legal implications of such an endeavor.

This was a closed meeting with 16 in-room participants and two on-phone participants. The format was a discussion forum. Therefore this report will be divided into sections based on topics of discussion detailing the key output of that discussion.

### What do we want from samples?

Prior to discussion regarding what types of samples and what we wanted to get from them, Jeff Gordon suggested and it was agreed that it was necessary to identify the principal stakeholders. These were primarily identified as carbon cycle and climate researchers, agricultural and human health organizations, fundamental science through the National Science Foundation (NSF), ecological & biodiversity research and interest groups (for which products must be developed as deliverables for the community). Rick Stevens also stipulated that there was potential commercial interest in novel functions or greater enzyme efficiency, which could be fed by data from this study.

Gordon also suggested that samples for the EMP should come from locations that capture the public imagination. For example, World Heritage Sites and sites of obvious human disturbance, such as EPA Superfund sites, would be ideal targets. Especially for the polluted or disturbed sites it was very important that we determine whether the environment can heal itself, or whether we need to identify roles for microbes in providing an anthropogenic solution to pollution. All participants agreed that cogent scientific rationales for the selection of sites would be crucial for the effort. The group went on to discuss how to capture public imagination in a similar way that the space program does. Gordon stipulated we need a grand challenge statement, as when President Kennedy said “Let’s send a man to the moon” in 1961. Ruth Ley highlighted that in order to excite people we needed to design extraordinary visuals. Jack Gilbert suggested that it was essential to ‘show’ microbial communities to the public.

Norman Pace said felt it very important that the EMP sample acquisition does not constitute ‘just-another-survey’. Rick Stevens noted that there is still huge interest in so-called microbial “dark matter”, meaning the unknown microbiome, which by definition needs to be explored. The global microbiome and its diversity is perhaps one of the largest questions within one of the most comprehensive dark-matter problems, because huge amounts of biodiversity remain unexplored. It was suggested that we have to be more quantitative about how we proceed. It may even be necessary to make some controversies about alternative projections. Physics is never short of making predictions about what is true and then having it confirmed. Are there conjectures we can make about diversity and then get this resolved through the scientific process? Noah Fierer suggested that physicists were much better organized, a group behavior we need to foster in the biological community. Noah went on to stipulate that microbiologists have an option to explore communities on the basis of pathogenicity and virulence, which could be used to excite the imagination.

Jack Gilbert went on to describe how to potentially select samples. It is necessary that the samples enable the production of a ‘topographical’ map of microbial function – which will be the most useful deliverable for the benefit of mankind. Rick Stevens stipulated that we can therefore use a phylogenetic survey to explore diversity and composition prior to targeted metagenomic (functional) discovery. Nikos Kyrpides suggested that maybe a phylogenetic profile was all that was necessary at this stage as we do not even know the distribution of microbes and this could be a primary goal in the short term.

### What data do we want associated with these samples?

It is essential that we have high quality environmental contextual data associated with every sample. Therefore we need a mechanism by which to ‘grade’ samples by the quality of their metadata. James Tiedje indicated that most soil samples collected to date have very poor metadata, although this was improving. Rob Knight indicated that through the work of organizations like the Genomic Standards Consortium (http://gensc.org) more samples would be collected with better metadata in the future. Janet Jansson suggested that the issue was standardization and quality assessment of metadata as well as sample quality. Janet pointed out that it is essential that we only collect high quality samples with high quality metadata. Norman Pace indicated that it is very important to get the chemistry of a sample collected. The group agreed to assess quality of potential metadata by requiring minimal information standards compliance, such as minimal information about a metagenomics sequence (MIMS) [2] and Minimal information about an Environmental Sequence (MIENS) [3]. Using the MIMS/MIENS standards compliance as a metric of quality would enable selection of only the highest quality samples in terms of comparability following analysis. It was agreed that this would be adopted as a core, objective criteria for sample prioritization within the EMP framework.

James Tiedje suggested that it was also extremely important to have information regarding the sample processing, e.g. soil researchers often only have air-dried samples, and often very little is known about the impact of these methods on downstream molecular analysis. Rob Knight argued that a role of the EMP could be to fund experiments to resolve the superstition regarding the impact of different sample preparation and experimental procedure on bias in the community analysis.

It will be absolutely necessary to have environmental parameters with all samples so that we can reduce the redundancy in sample analysis and explore a greater diversity of biomes for financial investment. Rick Stevens asked if there was a way to articulate classes of environments, i.e. parameter space for soils, marine ecosystems, lakes and rivers, etc. This information will help us to design a systematic way to sample environments, to enable a systematic march through the ways in which microbes live. Rachel Gallery indicated that NEON (http://www.neoninc.org/) chose different ecosystem types, including freshwater, to explore a diverse array of environments for long-term monitoring. They identified these by continental eco-regions. Environmental nomenclature was suggested as a method for choosing ecosystems, it was indicated that the ontology community had a vast range of tools to implement this, e.g. Habitat-Lite [4] for microbial ecosystems.

### Reaching out to the Scientific Community?

Jack Gilbert pointed out that we need to come up with an efficient model for sample acquisition, and went on to suggest that the EMP publish a short letter in every relevant journal highlighting the fact that we are looking for samples. Additionally, through advisory board members and involved parties we can ‘reach-out’ to the community through colleagues to identify excellent sample datasets. Rob Knight commented that the scientific community must drive sample collection, for example Margaret McFall-Ngai contributed a list of species for host-associated samples before the meeting. It is vital that we reach out to all microbial ecologists who have already collected samples that would have good metadata. Noah Fierer identified that Texas A&M have been collecting cow fecal samples, which is now at approximately 10,000 samples, and have excellent metadata. All these samples are frozen. Rachel Gallery suggested that the plant research community had many thousands of samples regarding plant pathology and endosymbiosis, which could be targeted. Jack Gilbert pointed out that Oliver Ryder had identified a wealth of host-associated samples from zoo animals in San Diego. Jim Tiedje defined one model, which would be to take all samples from all groups that meet the standards, from any ecosystems. This could represent a first pilot study, and could be done very soon. Rob Knight pointed out that this is very like the Community Sequencing Project from the Joint Genome Institute and we could implement that model. Norman Pace also agreed that this would be the most effective way of implementing a rapid development of the EMP.

Rick Stevens went on to discuss the importance of choosing an effective sequencing strategy for sample acquisition. He suggested that in order to verify samples from different researchers we would need to provide a standard sample per sequence run or DNA extraction. Noah Fierer suggested that we should select projects with no fewer than 100 samples; otherwise the economics would not work. Folker Meyer suggested that we should choose samples from ecosystems and biomes that have not yet been analyzed using this technology. It would be up to the advisory board to determine which samples should be analyzed.

Overall, it was agreed that the board represents a broad range of communities and that each person should ‘reach-out’ to their community and identify the types of projects that are available or will become available. This information should then be added to a central repository called the Global Environmental Sample Database (GESD) which will be used to grade, refine and select the environments for a series of pilot studies and subsequent analyses.

### How do we collect the samples?

Sample collection or acquisition was identified as the biggest problem. Rob Knight suggested that we need an infrastructure to collect sets of related samples that answer a specific biological question in a way that can be generalized to make larger-scale predictions, and to insure good quality samples and associated metadata. He went on to point out that it is vital that we recruit people to the EMP who are excited about contributing quality samples. However, the focus should first be on hypotheses testing, answering the central EMP questions. Jack Gilbert suggested that we should consider whether to accept samples or possible just DNA. Rob Knight noted that if we are to select DNA we need some standards for DNA quality. Additionally, we should consider sending primers to determine whether the DNA can be amplified prior to sending to the EMP. Folker Meyer suggested that we should only select projects that have demonstrated the ability to get good sequence data from Illumina platform from the samples they are sending. It was generally agreed that at this stage this would be overly difficult for the majority of research groups. Rick suggested that it would be essential to implement a standard sampling protocol for all environments now. One for each would be practical. It was agreed that this would be ideal but also very difficult, requiring a consortium for each community to come together and implement and enforce these standards. James Tiedje pointed out that NEON is an ideal example of these standards, but that this was not necessarily the biggest problem. Obtaining samples from outside of the US would be the most significant problem, licenses and permits would be required, and the countries from which samples were sent would need to agree to sequencing and downstream analysis to prevent litigation. Rick Stevens suggested that the EMP could potentially ship a sequencer into the country and this would prevent shipping costs and permits for the physical samples or DNA. Rob Knight suggested that one possible solution would be to have visitors come and extract samples at an EMP affiliated Laboratory.

### Ownership of samples and data?

James Tiedje pointed out that probably half he people will not participate in the EMP, because they would fear that they will lose control of their own samples and data. Rob Knight noted that the EMP needed to educate people to the fact that the EMP as a network will enable researchers to do more than trying to analyze the data in isolation, as is the current practice. Janet Jansson suggested that people will be amenable if they are guaranteed to have publication rights. Jack Gilbert suggested that we needed to get over the ‘bio-ego’ that is pervasive in our community. It is essential that this is done in an open and collaborative way, and by doing so they will have access to a more comparable and complete dataset than ever before. James Tiedje agreed that the ego was a big problem, and possibly insurmountable; if the EMP is successful as a pilot project and it can prove that the data generated is more effective than data in isolation, then the community will be more receptive. Frank-Oliver Glöckner suggested that people may still not buy into the bigger vision, this is an issue of trust and people will fear the autocracy of the EMP. James Tiedje said that in other disciplines this is not such a problem, for example the human genome publication all met together and participated in project with 125 authors. Jack Gilbert pointed out that people tend to trust the ‘village elders’ of a community, and if they support the EMP publically then people will follow.

### Moving forward?

Nikos Kyrpides suggested that we should work towards a NASA-style institute that binds us all together under the EMP umbrella. However, in the short term, the EMP requires a pilot study to demonstrate the benefit of this collaborative and comparable research initiative. Jack Gilbert suggested that the EMP should use a 16S ribosomal RNA gene survey to produce a map of 100,000 samples, and then select a range of samples for ultra-deep sequencing. Jack also noted that Trina McMahon has already signed on and provided an extensive series of temporal and biogeographic samples from temperate lakes, additionally Argonne National Laboratory also has a total of ~8000 samples ready to go for a broad 16S rRNA gene study which will be used to target metagenomics. Rob Knight asked the board to start making a compilation of other sample collections that they had access to. Janet Jansson pointed out that a large number of samples had already been sequenced, and that another approach would be to start compiling comparable metagenomic and 16S rRNA gene datasets to show the value of a centralized comparable network. Rob Knight and Folker Meyer pointed out that we need to make the data and analysis freely available to everyone. This must be as open as possible and contain a significant educational element. Dawn Field and Janet Jansson suggested that school outreach programs would be very effective. Rob Knight summarized the meeting by stipulating that we will try to demonstrate the value of the EMP initially with in-house samples. The board will reach out to networks for existing samples, such as Terragenome, Terra-Oceans and NEON, and solicit additional samples through professional contacts. The EMP should also work on a special Science Issue sponsored by Illumina and MoBio through which we advertise the EMP and promote community integration.
